# Farnesol Protects against Cardiotoxicity Caused by Doxorubicin-Induced Stress, Inflammation, and Cell Death: An In Vivo Study in Wistar Rats

**DOI:** 10.3390/molecules27238589

**Published:** 2022-12-06

**Authors:** Abdulrab Ahmed M. Alkhanjaf, Md Tanwir Athar, Zabih Ullah, Abdullah Mohammed H. Alsayhab, Ahmad Umar, Ibrahim Ahmed Shaikh

**Affiliations:** 1 Department of Clinical Laboratory Sciences, College of Applied Medical Sciences, Najran University, Najran 11001, Saudi Arabia; 2 College of Dentistry and Pharmacy, Buraydah Colleges, Buraydah 51452, Saudi Arabia; 3 Regional Laboratory, General Directorate of Health Affairs in Najran, Ministry of Health, Najran 66255, Saudi Arabia; 4 Department of Chemistry, Faculty of Science and Arts, Najran University, Najran 11001, Saudi Arabia; 5 Department of Pharmacology, College of Pharmacy, Najran University, Najran 64462, Saudi Arabia

**Keywords:** farnesol, reactive oxygen species, caspase-3, NF-kB, inflammation, cardioprotection

## Abstract

Doxorubicin (DOXO) is an antineoplastic drug that is used extensively in managing multiple cancer types. However, DOXO-induced cardiotoxicity is a limiting factor for its widespread use and considerably affects patients’ quality of life. Farnesol (FSN) is a sesquiterpene with antioxidant, anti-inflammatory, and anti-tumor properties. Thus, the current study explored the cardioprotective effect of FSN against DOXO-induced cardiotoxicity. In this study, male Wistar rats were randomly divided into five groups (*n* = 7) and treated for 14 days. Group I (Control): normal saline, p.o. daily for 14 days; Group II (TOXIC): DOXO 2.4 mg/kg, i.p, thrice weekly for 14 days; Group III: FSN 100 mg/kg, p.o. daily for 14 days + DOXO similar to Group II; Group IV: FSN 200 mg/kg, p.o. daily for 14 days + DOXO similar to Group II; Group V (Standard): nifedipine 10 mg/kg, p.o. daily for 14 days + DOXO similar to Group II. At the end of the study, animals were weighed, blood was collected, and heart-weight was measured. The cardiac tissue was used to estimate biochemical markers and for histopathological studies. The observed results revealed that the FSN-treated group rats showed decrease in heart weight and heart weight/body weight ratio, reversed the oxidative stress, cardiac-specific injury markers, proinflammatory and proapoptotic markers and histopathological aberrations towards normal, and showed cardioprotection. In summary, the FSN reduces cardiac injuries caused by DOXO via its antioxidant, anti-inflammatory, and anti-apoptotic potential. However, more detailed mechanism-based studies are needed to bring this drug into clinical use.

## 1. Introduction

Doxorubicin (DOXO), belonging to the anthracycline family, is among the most effective antineoplastic drugs ever developed for oncology. DOXO has been found to be highly effective in cancer chemotherapy, with notable success and well-known mechanisms for treating hematological and solid malignancies [1]. However, the use of DOXO is limited due to its major dose-dependent cardiotoxicity [2]. Cardiomyopathy induced by DOXO is exponentially increasing as more and more patients are being treated for cancer [1,3]. The mechanism of DOXO-induced cardiotoxicity is multifactorial in origin and involves complex pathways involving inflammation, oxidative stress, and apoptosis [4].

Increased oxidative stress and persistent increase in reactive oxygen species (ROS) stimulate lipid peroxidation and cause oxidative damage to myocytes, mitochondria, and cell membranes, leading to cardiotoxic manifestations [1]. After exposure to DOXO, oxidative stress is increased, leading to the expression of various transcription factors, such as nuclear factor kappa B (NF-κB), that further activate nucleotide-binding oligomerization domain-NOD-Like Receptor Protein 3 (NLRP3 inflammasome) [5]. This causes an increment in the release of proinflammatory cytokines from the myocardium, such as interleukin-1 beta (IL-1β) and tumor necrosis factor-alpha (TNF-α) [5]. In the pathogenesis of DOXO-induced cardiotoxicity, evidence shows that extrinsic and intrinsic pathways are involved. DOXO triggers cell apoptosis through two mechanisms, (1) by localizing directly into the mitochondria or (2) by increased calcium accumulation in cells and an increase in oxidative stress [6]. These mechanisms stimulate the release of cytochrome C, which further triggers apoptosis and causes cardiotoxicity [7].

Thus, looking into the complex and multifactorial mechanism of cardiotoxicity, more rigorous approaches are needed to prevent and reduce DOXO-induced cardiotoxicity and for its effective use in chemotherapy in clinical settings.

Recently, natural products such as essential oils have been extensively explored for their possible pharmacological effect, especially for their cardioprotective potency [8,9]. Chemically, farnesol (FSN) is an alcoholic sesquiterpene used in the cleansing and cosmetic industry and which is generally considered safe [9]. Moreover, FSN is approved by the FDA for consumption by humans as a flavoring agent [10]. In terms of the pharmacological attributes of FSN, it has been reported with anti-inflammatory, antioxidant, anticancer, anti-hypertensive, anti-arrhythmic, and hepatoprotective effects [11,12,13]. Based on the previous report on the cardioprotective potency of FSN, until now it has been explored only for the anti-arrhythmic, anti-hypertensive, and hypertrophic model of isoproterenol and aortic perfusion, preferably where cardiac contractility, echocardiographic and morphometric parameters such as L-type Ca^2+^ currents, left developed ventricular pressure, coronary pressure, and changes in ventricular action potential have been evaluated [14,15,16,17,18].

Until now, FSN has not been explored against DOXO-induced cardiotoxicity and for its anti-inflammatory effect. Hence, in the present study, we have aimed to evaluate FSN’s anti-inflammatory and cardioprotective potency against DOXO-induced cardiotoxic manifestations.

## 2. Materials and Methods

### 2.1. Drugs and Chemicals

DOXO (Adriamycin) was obtained from Pfizer, New York, NY, USA. Farnesol and Nifedipine were procured from Sigma Aldrich (St. Louis, MO, USA). ELISA kit for TNF-α, IL-10, IL-6, IL-1β were procured from Krishgen Biosystems, Mumbai, India. All other chemicals and reagents used in the experiment were of analytical grade.

### 2.2. Animals

42 Wistar albino rats were obtained from a reliable animal facility for experimental animals in Abha, Saudi Arabia. Najran University’s Scientific Ethical Committee approved the project, and a certificate of ethics approval was granted with the reference number: 443-42-59216-DS. All investigations were carried out in conformity with internationally established standards for the ethical treatment of animals (National Institutes of Health Publications No. 8023, revised 1978). Animals were kept in an animal house facility maintained at 25 ± 2 °C and with a 12-h dark-light cycle. Animals were fed with rat feed and water ad libitum. Rats were kept in the animal house for two weeks before experiments.

### 2.3. Dosing Paradigm

**Figure 1:**
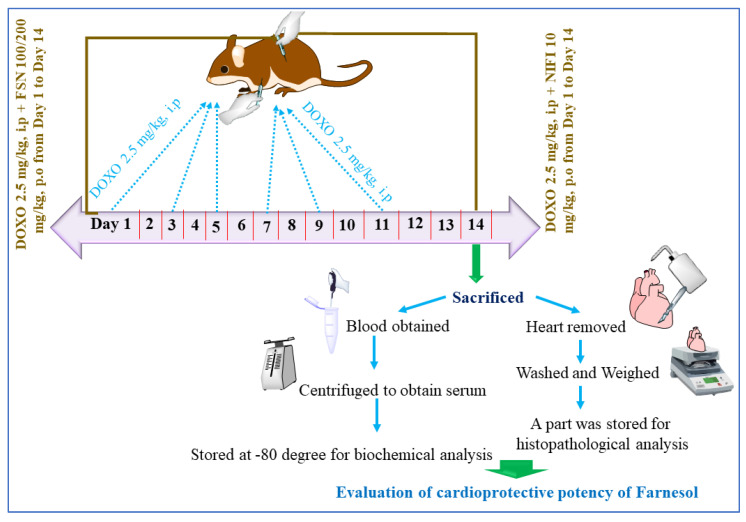
Showing the experimental design used in the study.

Thirty-five male Wistar rats (180–200 g) were divided into 5 groups (*n* = 7). The different groups received the treatment as follows:

Group I (control): normal saline, p.o. Daily for 14 days.
Group II (TOXIC): DOXO 2.4 mg/kg, i.p, thrice weekly for 2 weeks.
Group III: FSN 100 mg/kg, p.o. daily for 14 days + DOXO, similar to Group II
Group IV: FSN 200 mg/kg, p.o. daily for 14 days + DOXO, similar to Group II
Group V (Standard): nifedipine 10 mg/kg, p.o. Daily for 14 days + DOXO, similar to Group II. The graphical presentation of the treatment protocol is shown in Figure 1 [15,17].

### 2.4. Serum and Tissue Preparation

On day 14, before sacrifice, animals were accurately weighed and anesthetized using a mixture of ketamine and xylazine (100 mg/kg and 10 mg/kg, respectively). Blood was collected by the cardiac puncture method. Collected blood was centrifuged at 3000 rpm for 10 min, and serum was obtained and stored at −80 °C for biochemical analysis. Later, animals were sacrificed using the carbon dioxide euthanasia method, and the animal hearts from all the groups were removed, washed with ice-cold water, and weighed. Sections of the heart were cut and stored in formalin for biochemical analysis, whereas the remaining part was stored for histopathological analysis. Tissues of the heart were cut into two transverse sections. The basal part was frozen using dry ice. Later, this section was used for tissue homogenization using 0.1 M phosphate buffered saline at a pH of 7.4 and processed in the liquid processor with high-intensity ultrasonic waves. These mixtures were further centrifuged at 4 °C, and the supernatant obtained was used to determine the tissue markers [19,20].

### 2.5. Estimation of the Markers of Oxidative and Nitrative Stress

Markers for oxidative stress were estimated in the cardiac tissue. The level of lipid peroxidation was evaluated according to the method of Okhawa et al., 1979, in which the malondialdehyde (MDA) level signifies the extent of lipid peroxidation [21]. For this, tissue homogenates were centrifuged, and the obtained supernatant was adequately mixed with the Tris-HCL and incubated for 120 min. The resulting mixture was centrifuged at 1000 rpm for 10 min, and further trichloroacetic acid (10%) was mixed into it; absorbance was recorded at 540 nm and represented as MDA/mg protein [22]. The enzymatic activity of superoxide dismutase (SOD) was evaluated according to the previously published method by Marklund and Marklund, 1974 [23]. For this, the obtained supernatant was mixed with Tris-HCL and pyrogallol, and the absorbance was recorded at 420 nm. SOD activity was expressed as U/mg protein [24]. Catalase (CAT) activity was evaluated according to the previously mentioned method, and the absorbance was recorded at 240 nm; values were expressed as nmol H_2_O_2_/min/mg protein [25]. The activity of glutathione (GSH) was estimated according to the method of Sadlak and Lindsay, 1968, and the absorbance was recorded at 412 nm; values were expressed as µmol/mg of protein [25,26].

### 2.6. Estimation of Cardiac Injury Markers (LDH, CK-MB, BNPc, cTn-T and AST, ALT)

Markers of cardiac injury were estimated by determining the serum level of lactate dehydrogenase (LDH), creatine kinase-MB (CK-MB), cardiac Troponin-T (cTn-T), B-type natriuretic peptide (BNP), aspartate aminotransferase (AST), and alanine transaminase (ALT) on day 14 with the help of standard assay kits using the auto analyzer and as per the manufacturer’s instructions; the measured values were presented in IU/L. Specific biomarkers of cardiac injury, such as cardiac troponin-I and T serum levels, were measured by ELISA kits purchased from Krishgen Biosystems, Mumbai, India. Values were represented in ng/mL [27].

### 2.7. Examination of Cardiac TNF-α, IL-6, IL-10, and IL-1β Levels

Inflammatory markers were measured with the help of commercial kits based on enzyme-linked immune sorbent assay (ELISA). TNF-α, IL-6, IL-10, and IL-1β levels in the heart tissue were estimated using an ELISA kit for rats. Additionally, the level of nitric oxide (NO) activity in the heart tissue was also determined using an ELISA kit for rats [28].

### 2.8. Cardiac Apoptosis Examination

The activity of caspase-3 was assessed using ELISA kits for rats procured from Krishgen Biosystems, Mumbai, India, and performed as per the manufacturer’s instructions [28].

### 2.9. Histopathological Assessment

The tissue sections were fixed for 48 h in 10% neutral buffer formalin. These samples were dehydrated by passing alcohol, cleared with xylene to eliminate alcohol, and finally fixed and hardened in paraffin. Later these blocks were cut into thin 5-μM sections with a microtome and then allowed to float in a water bath. These floating sections were mounted onto microscope slides, air dried at 60 °C for 20 min in an oven, and stained with H&E (hematoxylin-eosin) dye to examine any histopathological changes using light microscopy (Olympus CX31) [28].

## 3. Statistical Analysis

One-way ANOVA (Tukey’s multiple comparison test) was used for the statistical analysis. *p* < 0.05 was considered significant. Values were presented as Mean ± SEM of different groups.

## 4. Results

### 4.1. Effect of FSN on DOXO-Induced Change in Body and Heart Weight

Administration of DOXO (TOXIC group) caused a significant reduction in body weight and increment in heart weight and ratio of HW/BW compared to the control group, which signifies cardiac hypertrophy. When the animals were treated with FSN 100 and 200 mg/kg, p.o, a dose of 200 mg/kg, p.o showed a reversal in the change in body weight and heart weight and the ratio of HW/BW as compared to DOXO. Moreover, animals treated with the standard (NIFI 10 mg) fully reversed the HW/BW ratio towards normal, as shown in Table 1.

### 4.2. Effect of FSN on DOXO-Induced Cardiac Injury Markers (LDH, CK-MB, cTn-T, and BNP)

**Figure 2:**
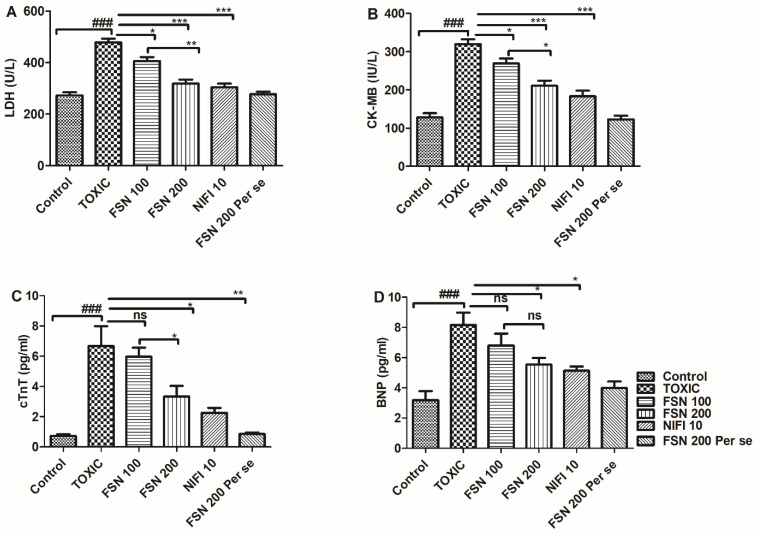
Showing the effect of FSN on DOXO-induced altered cardiac injury markers, lactate dehydrogenase (LDH) (**A**), creatine kinase-MB (CK-MB) (**B**), cardiac Troponin-T (cTn-T) (**C**) and B-type natriuretic peptide (BNP) (**D**). ^###^ *p* < 0.001 significant versus control; * *p* < 0.05, ** *p* < 0.01, *** *p* < 0.001 significant versus TOXIC and ns is non-significant versus TOXIC.

Administration of DOXO caused a significant (*p* < 0.001) elevation in the level of cardiac-specific markers, and thus validated cardiac injury as compared to the control group. When the animals were treated with FSN 100, the levels of CK-MB and LDH showed a significant reduction, whereas a nonsignificant effect was observed against cTn-T and BNP. Similarly, treatment with FSN 200 and NIFI 10 significantly reduced CK-MB, LDH, cTn-T, and BNP levels. Moreover, when we compared the effect between FSN 100 and FSN 200, a significant difference was observed for LDH, CK-MB, and cTnT, whereas no significant difference was observed for BNP. However, upon comparing the cardioprotective effect of FSN 200 and NIFI 10, NIFI 10 showed numerically higher cardioprotection, as shown in Figure 2. Notably, FSN 200 per se showed similar changes in the level of cardiac injury markers, as exhibited by the control group. Hence, the safety of FSN 200 was also validated.

### 4.3. Effect of FSN on DOXO-Induced Cardiac Injury Markers (ALT and AST)

**Figure 3:**
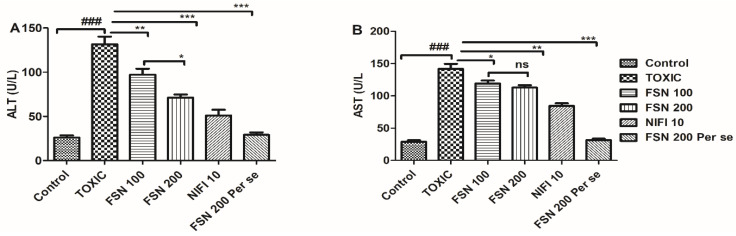
Showing the effect of FSN on DOXO-induced altered non-specific cardiac injury markers, aspartate aminotransferase (AST) (**A**), and ala-nine transaminase (ALT) (**B**). ^###^ *p* < 0.001 significant versus control; * *p* < 0.05, ** *p* < 0.01, *** *p* < 0.001 significant versus TOXIC and ns is non-significant versus TOXIC.

The administration of DOXO caused a significant elevation in ALT and AST levels compared to the control group. When the animals were treated with FSN 100, ALT and AST levels showed mild reduction compared to the DOXO-treated group. Similarly, treatment with FSN 200 and NIFI 10 significantly reduced ALT and AST levels. Upon comparing the cardioprotective effect of FSN 200 and NIFI 10, NIFI 10 showed numerically higher cardioprotection, as shown in Figure 3. Moreover, when we compared the effect between FSN 100 and FSN 200, a significant difference was observed for ALT, whereas no significant difference was observed for AST. Notably, FSN 200 per se showed a similar change in the level of liver injury markers, as exhibited by the control group. Hence, the safety of FSN 200 was also validated.

### 4.4. Effect of FSN on DOXO-Induced Oxidative and Nitrative Stress

**Figure 4:**
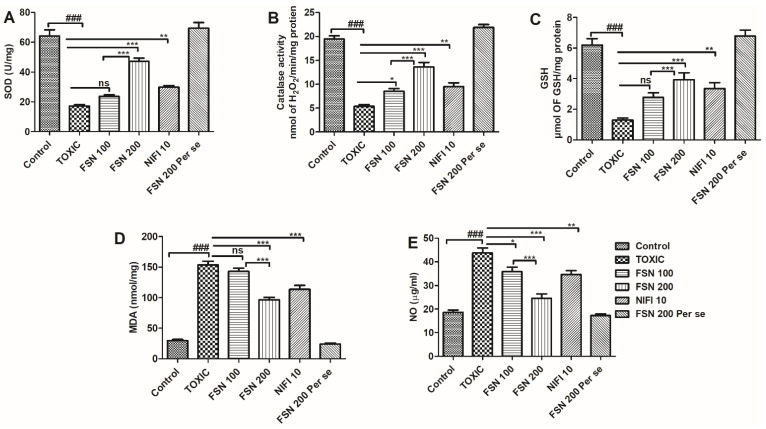
Showing the effect of FSN on DOXO-induced oxidative and nitrative stress enzymes superoxide dismutase (SOD) (**A**), Catalase (**B**), glutathione (GSH) (**C**), malondialdehyde (MDA) (**D**) and nitric oxide (NO) (**E**). ^###^ *p* < 0.001 significant versus control; * *p* < 0.05, ** *p* < 0.01, *** *p* < 0.001 significant versus TOXIC, and ns is non-significant versus TOXIC.

Administration of DOXO caused a significant elevation in the level of TBARS and NO, whereas, it reduced the enzymatic activity of SOD, CAT, and antioxidant level of GSH compared to the control group and hence showed oxidative and nitrative stress. When the animals were treated with FSN 100, no significant difference in the level of TBARS, and a mild reduction in the level of NO and elevation in the enzymatic activity of CAT, were observed compared to the DOXO-treated group. However, FSN 100 showed a mild increment in the enzymatic activity of SOD and level of GSH as compared to the DOXO-treated group. However, treatment with FSN 200 and NIFI 10 showed a significant reduction in MDA, TBARS, and NO levels. It reversed the enzymatic activity of SOD, CAT, and antioxidant levels of GSH towards normal. Moreover, when we compared the effect between FSN 100 and FSN 200, a significant difference was observed for SOD, CAT, GSH, TBARS, and NO. However, upon comparing the antioxidant potential and cardioprotective effect of FSN 200 and NIFI 10, FSN 200 showed a numerically higher antioxidant effect, as shown in Figure 4. Notably, FSN 200 per se showed a similar change in the markers of oxidative stress, as exhibited by the control group. Hence, the safety of FSN 200 was also validated.

### 4.5. Effect of FSN on DOXO-Induced Cardiac Inflammation and Apoptosis

**Figure 5:**
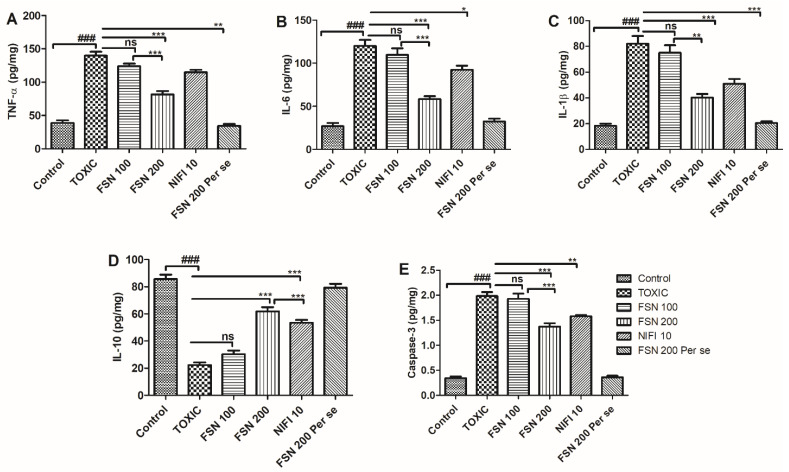
Showing the effect of FSN on DOXO-induced elevation of proinflammatory cytokines TNF-α (**A**), IL-6 (**B**), IL-1β (**C**), decrease in cytokines IL-10 (**D**) and increase in apoptotic marker Caspase-3 (**E**). ^###^ *p* < 0.001 significant versus control; * *p* < 0.05, ** *p* < 0.01, *** *p* < 0.001 significant versus TOXIC, and ns is non-significant versus TOXIC.

Administration of DOXO caused a significant elevation in the level of proinflammatory cytokines (TNF-α, IL-6, and IL-1β), whereas it reduced the level of anti-inflammatory cytokines (IL-10) when compared to the control group and hence showed cardiac inflammation. Similarly, DOXO treatment showed an increased level of caspase-3 compared to the control group and the cardiac apoptosis that cumulatively led to cardiotoxic manifestations. When the animals were treated with FSN 100, a mild reduction in the level of TNF-α was found, whereas no significant difference in the level of IL-6, IL-1β, IL-10, and caspase-3 was found compared to the DOXO-treated group. However, treatment with FSN 200 and NIFI 10 showed a significant reduction in the level of TNF-α, IL-6, IL-1β, and caspase-3 and an increased level of IL-10. However, upon comparing the anti-inflammatory and cardioprotective effects of FSN 200 and NIFI 10, FSN 200 showed numerically higher anti-inflammatory effects, as shown in Figure 5. When we compared the effect between FSN 100 and FSN 200, a significant difference was observed for TNF-α, IL-6, IL-1β, IL-10, and caspase-3. Notably, FSN 200 per se showed similar changes in the level of cardiac inflammation and apoptosis markers, as exhibited by the control group. Hence, the safety of FSN 200 was also validated.

### 4.6. Effect of FSN on DOXO-Induced Histopathological Aberrations

**Figure 6:**
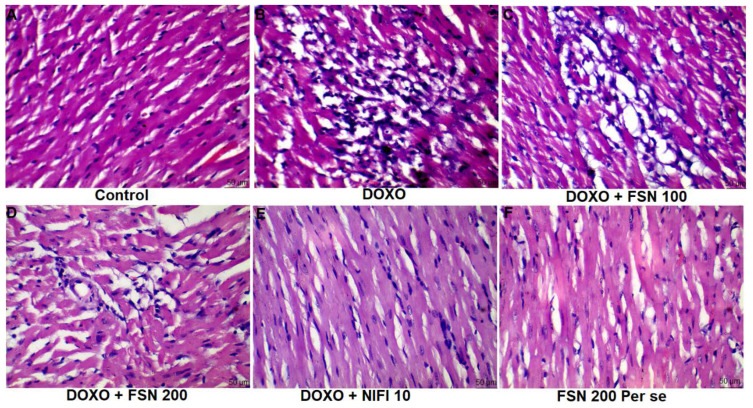
(**A**–**F**), showing the effect of FSN on DOXO-induced histopathological abrasions in the cardiac tissue. DOXO treated group showed marked histopathological damage such as cellular disintegration, vacuolation, pyknosis and myofibrillar damage. Treated with FSN 200 and NIFI 10 significantly reduced the histomorphological damage whereas FSN 100 was found to be ineffective [scale bar 50 µm]. FSN 200 per se showed similar histopathological attributes as those exhibited by the control group. Hence, the safety of FSN 200 was also validated.

Based on the histopathological analysis, the normal control group showed well-organized myocardial tissue with no sign of cellular integration, pyknosis, hemorrhage, or macrophagic/lymphocytic infiltrate. However, in the DOXO-treated group (TOXIC), myocardial disintegration, pyknotic nucleus, and damaged cellular architecture were found. Treatment with FSN 100 showed no significant improvement against DOXO-induced structural damage. In contrast, FSN 200 and NIFI 10 considerably reversed the histomorphological aberrations towards normal and showed a cardioprotective effect, as shown in Figure 6. Notably, FSN 200 per se showed similar histopathological attributes as those exhibited by the control group. Hence, the safety of FSN 200 was also validated.

## 5. Discussion

Based on the recently published evidence, the incidence and prevalence of cancer are increasing exponentially, and so is the use of various chemotherapeutic drugs [29]. DOXO is an extensively used anticancer drug that has significantly increased patients’ survival duration. However, cardiotoxic manifestation as a side effect of DOXO is a major limitation of its use. Scientific evidence has shown acute and chronic cardiotoxicity caused by DOXO [3,4]. Thus, the FDA has approved dexrazoxane as an adjuvant to prevent DOXO-induced cardiotoxicity [30]. However, apart from this adjuvant, no other drug is available to take care of this situation.

Moreover, in recent times, natural bioactive compound use has increased extensively because of its multifactorial mechanisms of action [31,32,33]. Thus, various attempts are being made to explore natural products’ cardioprotective potency so that they can be used as an adjuvant. In line with that, we have explored the cardioprotective potential of FSN against DOXO-induced cardiotoxic manifestations. Since DOXO causes significant cardiac inflammation and apoptosis, we tried to generate scientific evidence of FSN for its cardioprotective potential. Data from this study proved that treatment with FSN ameliorated the cardiac injury induced by DOXO, reduced inflammation and apoptosis, and restored the antioxidant capacity, pointing toward the protective effects of FSN against DOXO-induced cardiac injury [15,16,17,18]. This study highlights the therapeutic advantages of FSN when it will be combined with DOXO-like anticancer agents.

In the current study, treatment with DOXO for 14 days induced an increase in heart weight as well as HW/BW ratio. The increase in heart weight may be attributed to the enlarged, dilated, and hypertrophic atrium and ventricles [34,35,36,37,38]. After 14 days of treatment with FSN, results proved that FSN partly or fully inhibited the tissue apoptosis/necrosis process, thus preventing myofibrillar and cardiomyocyte loss and ameliorating the increase of the heart weight (Table 1). Our findings corroborate with the findings of de Souza et al., 2020, who reported that FSN significantly reversed isoproterenol-induced pathological cardiac hypertrophy in rats [14].

Specific and non-specific markers of cardiac injuries, such as LDH, CK-MB, cTn-I, BNP, and cTn-T, are released from the cardiac tissue when myocardial cells become damaged due to necrosis and the permeability of the cell membrane increases. Because cardiac tissue damage happens in response to toxic exposure, these enzymes enter the bloodstream, thus increasing their concentration in the serum [8,39]. Thus, serum enzymes such as CK-MB, BNP AST, ALT, and LDH are known and well-validated diagnostic markers for cardiotoxicity [38]. Troponin-T and Troponin-I are cardiac-specific troponins involved in myocardial cell injury [40]. In the current study, increased expression of ALT, AST, CK-MB, LDH, and increased levels of cTnT and cTnI were seen in the DOXO treatment group. When animals were treated with FSN, a dose-dependent reduction in the serum level of these cardiac injury markers was found, which signifies the cardioprotective potential of FSN. We thus concluded that a decrease in the activity of these enzymes was noted in test group animals (FSN-treated) which caused a reduction in the myocardial damage induced by DOXO, thus restricting the leakage of enzymes into the bloodstream; this proves the protective effect of FSN on myocardial tissues.

The cardiotoxic role of persistent oxidative and nitrative stress is well-established in various preclinical and clinical studies. Several cardiotoxic agents exhibit cardiotoxicity via inducing oxidative and nitrative stress, whereas cardioprotective agents showed its effect via scavenging these free radicals [41,42,43]. Considering these facts, in the present study, DOXO administration caused a significant increase in lipid peroxidation, exhibited by distinct elevations of TBARS, along with an increase in the level of NO [42]. Increased ROS have been reported to cause oxidation of myocardial lipid component that results in the increased level of TBARS. Increased TBARS on the one hand aggravates the ROS production, whereas on the other hand it increases the production of proinflammatory cytokines such as COX-2, TNFα, and IL-6, to name a few [44,45]. Moreover, DOXO administration also reduced the enzymatic activity of SOD, CAT, and antioxidant level of GSH that further aggravates oxidative stress [42]. When the animals were treated with FSN 100, FSN 200, and NIFI 10, FSN 200 showed the most potent antioxidant activity, followed by the NIFI 10. In contrast, FSN 100 failed to reverse the derailed level of MDA, NO, and other antioxidant enzymes to normal.

Apart from cardiac oxidative stress, cardiac tissue inflammation exhibited by DOXO is yet another major pathological attribute that leads to cardiotoxic manifestations [46]. DOXO administration leads to an increase in NF-κB expression and stimulates its nuclear translocation. Mechanistically, exposure of DOXO directly or DOXO-induced oxidative causes phosphorylation of iκB that withdraws the inhibitory action from NFκB for nuclear translocation, hence causingNFκB to undergo nuclear translocation. In the nucleus, it binds on the specific DNA sequence and regulates the transcription of various pro-inflammatory cytokines [47], such as TNF-α, IL-1β, and IL-6 [8,48]. Moreover, DOXO-induced oxidative stress and increased lipid peroxidation trigger the inflammatory reaction and cause activation of proinflammatory cytokines [8]. Apart from modulation of cytokine production, DOXO administration also increases the activity of transforming growth factor beta (TGF-β) and p38 mitogen-activated protein kinases, and affects nuclear factor erythroid 2–related factor 2, heme oxygenase–1 pathways, and cJUN pathways, leading to cardiotoxic manifestations. FSN possesses strong antioxidant and anti-inflammatory potential in various published reports [49]. Considering these facts, in the present study, DOXO administration caused a significant increase in the level of proinflammatory cytokines. When the animals were treated with FSN 100, FSN 200, and NIFI 10, FSN 200 showed the most potent anti-inflammatory activity, followed by NIFI 10. In contrast, FSN 100 failed to reverse the derailed cardiac inflammation toward normal.

One of the mechanisms of DOXO-induced cardiotoxicity is stimulation of cardiomyocyte apoptosis via the activation of caspase-3 [50]. The mechanism of cardiac apoptosis is the direct consequence of DOXO and the indirect effect of DOXO-mediated oxidative stress and inflammation [50]. Mechanistically, exposure to DOXO directly or indirectly causes DOXO-induced oxidative stress and inflammation, which affects the calcium homeostasis via endoplasmic reticulum and mitochondrial stress. This leads to increase levels of calcium that damage the mitochondrial membrane integrity, followed by the discrete release of cytochrome C. Increased cytochrome C then regulates the formation of caspase-3 via apoptosome and ultimately causes myocardial apoptosis [51,52,53]. According to previous reports, FSN has shown modulation of calcium homeostasis leading to cardioprotection. Thus, in our present study, we also evaluated the antiapoptotic potential of FSN. A significant elevation in the level of caspase-3 was found and confirmed the DOXO-mediated apoptosis. When the animals were treated with FSN 100, FSN 200, and NIFI 10, FSN 200 showed the most potent antiapoptotic activity, followed by the NIFI 10. In contrast, FSN 100 failed to reverse the myocardial apoptosis towards normal. These findings are consistent with the previous reports that FSN decreased the ERK1/2, Bax, and caspase-3 activation and increased AKT and Bcl-2 protein expression, which could be associated both with the pathological cardiac remodeling and with cardioprotection [11,12,13,14].

Histopathological aberrations are always considered concrete evidence of cardiac toxicity [54]. Two weeks of DOXO treatment caused the myofibrillar loss, perinuclear vacuolization, myocardial swelling, and disarray of myocardial fibers with cytoplasmic vacuolization. These histopathological damages were significantly reversed with the FSN 200 and NIFI 10, thus restoring the structural integrity of the cardiomyocytes.

## 6. Conclusions

In summary, the results of this study showed that oral administration of FSN for a duration of 14 days decreased the extent of oxidative stress and nitrative stress and reduced the extent of myocardial damage via reducing the serum level of cardiac injury markers. Moreover, FSN treatment exhibited potent anti-inflammatory and anti-apoptotic effects, thus reversing the histopathological injury caused by DOXO. Meanwhile, FSN 100 showed negligible or mild cardioprotection compared to the FSN 200 and NIFI 10. The explanation for this could be the pharmacokinetic limitation of FSN, which failed to achieve the therapeutic window at the lower dose. Thus, based on the outcome of the present study, we conclude that FSN could be a potent cardioprotective agent, but pharmacokinetic limitations should be considered before bringing this agent from the bench to the bedside. We further suggest exploring the cardioprotective activity of FSN 100 in a nano carrier-based drug delivery system so that the existing pharmacokinetic limitations can be overcome and the maximum pharmacological effect can be obtained at a much lower dose.

## Figures and Tables

**Table 1:** Showing the effect of FSN on DOXO-induced change in HW/BW ratio.

Groups	Body Weight (in g)	Heart Weight (in mg)	HW/BW (mg/g)
Control	253.5 ± 4.12	839.9 ± 7.16	3.31 ± 0.04
TOXIC	230 ± 4.85	974.7 ± 5.34 ***	4.23 ± 0.03
FSN 100	248.3 ± 4.82	963.5 ± 7.12 ^ns^	3.88 ± 0.02
FSN 200	241.7 ± 3.07	855.4 ± 6.64 ^###^	3.53 ± 0.02
NIFI 10	245 ± 2.58	833.2 ± 2.18 ^###^	3.39 ± 0.03

FSN; farnesol, NIFI; Nifedipine. *** *p* < 0.001 compared to normal control group; ^###^ *p* < 0.001 compared to TOXIC group. ns is non-significant versus TOXIC.

## Data Availability

All data related to this research is presented in the article.

## Sample Availability

The sample is available with the corresponding author on reasonable requests fulfilling the criteria of the author institutions.
